# Establishing a clinical service for the treatment of osteoid osteoma using magnetic resonance-guided focused ultrasound: overview and guidelines

**DOI:** 10.1186/s40349-016-0059-6

**Published:** 2016-05-20

**Authors:** Michael J. Temple, Adam C. Waspe, Joao G. Amaral, Alessandro Napoli, Suzanne LeBlang, Pejman Ghanouni, Matthew D. Bucknor, Fiona Campbell, James M. Drake

**Affiliations:** The Hospital for Sick Children, University of Toronto, 555 University Avenue, Toronto, ON M5G 1X8 Canada; Sapienza University of Rome, Piazalle Aldo Moro, 5, Rome, 00185 Italy; University MRI Image Guided Therapy Center, 3848 Fau Blvd., Suite 200, Boca Raton, FL 33431 USA; Stanford University Medical Center, 1201 Welch Road, Room P267, Stanford, CA 94305 USA; UCSF School of Medicine, 513 Parnassus Ave., San Francisco, CA 94143-0410 USA

**Keywords:** Focused ultrasound, Therapy, Ablation, Bone, Osteoid osteoma, Clinical, Review, Guideline

## Abstract

Recent studies have demonstrated the effectiveness of magnetic resonance-guided focused ultrasound (MRgFUS) in the treatment of osteoid osteoma (OO), a painful, benign bone tumor. As MRgFUS is a noninvasive and radiation-free treatment, it stands to replace the current standard of care, percutaneous radiofrequency, or laser thermal ablation. Within an institution, creation of a clinical OO MRgFUS treatment program would not only provide cutting edge medical treatment at the current time but would also establish the foundation for an MRgFUS clinical service to introduce treatments currently under development into clinical practice in the future. The purpose of this document is to provide information to facilitate creation of a clinical service for MRgFUS treatment of OO by providing (1) recommendations for the multi-disciplinary management of patients and (2) guidelines regarding current best practices for MRgFUS treatment. This paper will discuss establishment of a multi-disciplinary clinic, patient accrual, inclusion/exclusion criteria, diagnosis, preoperative imaging, patient preparation, anesthesia, treatment planning, targeting and treatment execution, complication avoidance, and patient follow-up to assure safety and effectiveness.

## Background

Magnetic resonance-guided focused ultrasound is an emerging option in the treatment of osteoid osteoma. Early work in this area shows great promise for this noninvasive and radiation-free treatment modality [1–3]. The purpose of this document is to provide information to help facilitate creation of a clinical service for magnetic resonance-guided focused ultrasound (MRgFUS) treatment of osteoid osteoma (OO) by providing (1) recommendations for the multi-disciplinary management of patients and (2) guidelines regarding current best practices for MRgFUS treatment. This paper will discuss establishment of a multi-disciplinary clinic, patient accrual, inclusion/exclusion criteria, diagnosis, preoperative imaging, patient preparation, anesthesia, treatment planning, targeting and treatment execution, complication avoidance, and patient follow-up to assure safety and effectiveness.

Osteoid osteoma is a small, painful benign bone lesion that accounts for 10 % of all bone tumors [4]. OO can affect male and female patients as young as a few months, up to the elderly, but predominately affects males between 10 and 35 years of age. These lesions can be painfully disabling, cause neurological dysfunction due to spinal compression [5], be difficult to remove surgically, and may recur following excision [6]. OOs most commonly occur not only in the tubular long bones, such as the femur, tibia, and humerus [6–8], but can also be found in other bones such as the pelvis and spine [5].

Radiographically, the osteoid-rich nidus and surrounding vascular connective tissue typically appears as a central sclerotic focus (nidus) surrounded by a lucent area [4]. The nidus consists of interconnected trabeculae and woven bone and produces prostaglandin E2 and prostacyclin or prostaglandin I2 (PGE2 and PGI2), hormones that sensitize the surrounding tissue to pain [9].

Osteoid osteomas may resolve spontaneously but the process can take several years. For many patients, the pain can be severe, with significant impact on health-related quality of life, including effects on physical, emotional, social, and role functioning. Early pain management interventions can reduce pain duration, pain-related disability, and potentially healthcare expenditures.

Conventional treatment options involve the use of pain medication, surgical resection, or, more recently, percutaneous thermal ablation. Conservative management is often suboptimal, with analgesics providing inadequate pain relief and causing side-effects, e.g., intolerance from prolonged use of nonsteroidal anti-inflammatory drugs (NSAIDs). Surgical resection is invasive and associated with a risk of infection, bleeding, and fracture. Percutaneous treatment methods are less invasive and carry less risk than traditional surgery [6, 7]. However, bleeding, infection, and fracture are still possible [10]. Additional risks relate to the use of ionizing radiation for guidance and thermal diffusion. Burns of the needle tract and skin can result from heat transmitted backwards along an uninsulated coaxial introducer needle [11].

Minimally invasive, percutaneous thermal ablation techniques, such as computed tomography (CT)-guided radiofrequency (RF) ablation, laser ablation [7], and cryoablation [12] are the current standard-of-care treatment for peripheral bone lesions. However, the possibility of thermal injury to nontarget tissue is a concern with thermal ablation procedures. With CT-guided thermal ablation, tissue temperature distribution cannot be measured directly, placing nearby vital structures (e.g., arteries, spinal cord, peripheral nerves) at risk of injury [13–15]. RF ablation induces temperatures above 90 °C that are maintained for several minutes in order to thermally coagulate the target [6, 16, 17]. If the temperature rises above 100 °C, charring blocks RF signal transmission and further heating of the adjacent tissue is not possible. During laser ablation, up to 1200 J of energy is deposited [18, 19] with resultant temperatures exceeding those during RF. In some cases, the potential for nontarget thermal damage necessitates placement of a temperature sensing needle and the injection of carbon dioxide, air, or liquid to act as a heat sink, thereby increasing the complexity and invasiveness of the procedure.

Magnetic resonance (MR)-guided laser ablation has become an attractive alternative to CT-guided ablation due to the laser’s intrinsic MR compatibility and the ability to integrate real-time temperature monitoring into the procedure [18]. However, in order to improve patient access, these procedures are typically performed in open, low-field strength magnets that do not provide adequate signal to noise for accurate thermometry near low signal tissues, such as bone. Very few centers have adopted the use of MR guidance for percutaneous ablation.

A noninvasive treatment modality that provides the same efficacy as percutaneous ablation, monitors thermal energy dose during the treatment and reduces risk of infection and skeletal weakening would be advantageous.

## Magnetic resonance-guided high intensity focused ultrasound

MR-guided focused ultrasound is a noninvasive, outpatient treatment modality being used to treat uterine fibroids and painful bone metastases. Many ongoing studies are currently underway investigating its use for cancer and other diseases [20].

MRgFUS is a thermal treatment modality that uses high-power ultrasonic energy to thermally coagulate tissues in the body. A specially designed transducer is used to focus a beam of ultrasound energy into a small volume at a specific target site in the body. The transducer is placed extracorporeally and is aimed through the skin to the target without requiring an incision or a sterile operating environment. The treatment volume of a single sonication is small, roughly the size of a grain of rice. Manufacturers have worked to increase treatment volumes using electronic and mechanical approaches. In the current generation of machines, electronic steering allows treatment of up to 5.1 cubic centimeters (cc) and mechanical transducer motion increases the maximum treatment volume to 20 cc per sonication in soft tissue. Due to the sharp focus of the transducer, the ultrasound beam produces thermal ablation only in the target zone, which results in cell death when individual cells are exposed to temperatures above 57 °C for a duration of 1 s or more [21]. Meanwhile, minimal heating of the surrounding tissues (located within the ultrasound beam but not within the target zone) is harmless since normal tissue perfusion quickly dissipates the energy. For bone lesions, since there is no mechanical penetration, MRgFUS reduces the chance of pathologic fracture and infection.

MRgFUS heating of bone requires relatively little energy to achieve thermal ablation compared to soft tissue treatment, resulting in a wider safety margin. The energy required for MRgFUS heating of tissue varies due to a number of factors including the water content, density, heat capacity, thermal diffusion, and blood flow. Bone absorbs MRgFUS energy 50 times more readily than soft tissue does [22, 23]. This improved efficiency in part reflects the lower water content and increased acoustic attenuation of bone. Water has a very high specific heat capacity, requiring a large amount of energy to increase temperature. In addition, tissue perfusion and blood flow act as a heat sink in soft tissues, absorbing and removing heat energy from the surrounding tissues.

The potential for beam refraction and heat conduction are among the few limitations to the use of ultrasound therapy. The area of treatment must be acoustically accessible, i.e., there is an unobstructed soft tissue window between the targeted lesion and the MRgFUS transducer. The presence of a scar or an orthopedic implant could deflect the beam resulting in an off-target injury or skin burn. As with any thermal therapy, thermal diffusion could result in injury to vital structures (nerves, blood vessels, or the skin surface) in close proximity to the area of treatment. (See the complications and complication avoidance section below for specific recommendations).

**Fig. 1:**
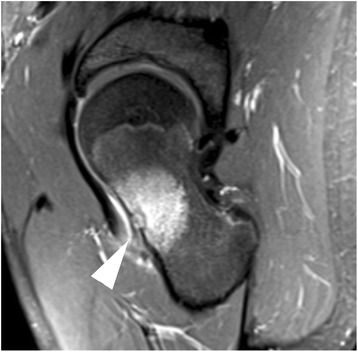
MR of osteoid osteoma. Sagittal T2-w fat saturated MRI image of a 1-cm cortical osteoid osteoma (*white arrowhead*) with prominent bone marrow edema surrounding the left femoral neck lesion. The location of the intraarticular lesion allows for an unobstructed treatment window (i.e., no nerves or other vital structures are present in the ultrasound path)

Magnetic resonance imaging (MRI) is used to provide the anatomical information necessary to safely guide and focus the ultrasound beam on the target (i.e., the lesion/nidus, and adjacent periosteum containing the nerves and vasculature for the nidus) and to perform real-time multi-planar thermal mapping [24], required to ensure therapeutic temperatures are produced in the target region and that surrounding healthy tissue structures are spared. See Fig. 1. Cell death from coagulative destruction of the lesion ends prostaglandin production. The mechanism of posttreatment pain relief, despite residual lesion perfusion, is unclear [2]. Hypotheses include thermal periosteal denervation and/or thermal ablation of the nidus’ vasculature that diminishes pressure on surrounding tissues or reduced prostaglandin production.

Treatment of osteoid osteoma with MRgFUS was initially performed in a small group of adolescent and adult patients (*N* = 6) by Napoli et al. [2]. An Insightec ExAblate 2100 machine was used to deliver a mean energy of 866 J (43 W acoustic power for an exposure duration of 20 s) to achieve thermoablative temperatures of 65 °C in the soft tissue immediately adjacent to the bone. These are comparable levels of energy applied during laser ablation procedures in bone [18, 19]. Following a single treatment, all of the patients in this study experienced complete pain response by 6-month follow-up, and there were no device-related serious adverse events (SAE) reported. Pain scores diminished from 7.9 ± 1.9 on an 11-point visual analog scale (VAS) to 0.0 ± 0.0. During follow-up imaging (1, 3, 6 months), edema and hyperemia associated with the osteoid osteoma gradually decreased in all treated lesions. The vasculature of the central nidus, however, continued to enhance under contrast-enhanced T1-weighted MRI in 67 % of patients, indicating that lesions were likely undertreated, even though the pain resolved. Previously, total ablation of the nidus was considered necessary to prevent any recurrent tumor. At 6-month follow-up, no patients required medication for bone pain and had no further follow-up treatments for pain recurrence.

A prospective, multi-institutional trial followed 29 patients undergoing MRgFUS of nonspinal osteoid osteoma [1]. All treatments were technically successful using 7 ± 3 sonications (1–12) and 1180 ± 736 J (416–3645) with an Insightec Exablate 2100 unit. At 1 year, 26/29 patients were pain free, consistent with a 90 % clinical success rate. The remaining 10 % (three patients) experienced partial pain relief. Two patients underwent subsequent radiofrequency ablation and one open surgical resection. There were no adverse events reported. The 90 % clinical success rate with MRgFUS OO treatment is equivalent to that of CT-guided radiofrequency ablation [25, 26].

Masciocchi et al. prospectively gathered data on 13 patients undergoing MRgFUS treatment and compared them to 30 propensity score-matched patients who underwent radiofrequency ablation [3]. The methods were found to be equivalent in regard to pain response and motor functional recovery.

MRgFUS has also been used to palliate the pain associated with metastatic bone cancer [27–31]. The phase III multi-center study by Hurwitz et al. [27] reported a positive pain response (decrease in pain score by 2 or more points by 3 months without an increase in pain medication) in 64 % (72/112) of patients treated with MRgFUS that were not candidates for initial or repeated radiation therapy.

## Clinical and technical approach to MRgFUS treatment

The following sections provide recommendations for establishing a MRgFUS clinic, patient work-up, and technical details of treatment.

### Team composition and multi-disciplinary clinic

MRgFUS treatment of osteoid osteoma requires the expertise of several disciplines including interventional radiology, orthopedic surgery, anesthesia (including a pain expert), radiography (MRI technologist), physics (MRgFUS physicist), and nursing. Once a team has been established, a multi-disciplinary clinic should be formed to screen potential patients in order to determine which patients are best suited for MRgFUS and to provide ongoing care and follow-up to previously treated patients. The multi-disciplinary clinic format enables efficient assessment of patients by interventional radiologists, orthopedic surgeons, and anesthesiologists (with expertise in both anesthesia and pain management) in a single visit.

### Patient selection

Proper selection of patients is a critical step to the successful execution of MRgFUS treatment of OO. In addition to using good medical judgment when seeking potential candidates for this therapy, Table 1 describes suggested inclusion and exclusion criteria. There may be patients who fall outside of these criteria who are still appropriate for treatment.

**Table 1:** General inclusion and exclusion criteria for osteoid osteoma MRgFUS treatment

*Inclusion criteria* • Age ≥5 years • Subject able to give informed consent or subject assents with informed consent from parent or guardian • Weight <140 kg (requirement to fit safely on top of the MRgFUS table and inside the MR magnet) • Definitive radiographic and clinical presentation of an osteoid osteoma o *OR* biopsy proven osteoid osteoma when clinical or imaging findings are inconclusive • Pain specifically at the site of interest (target lesion) • Pain intensity (for the target lesion) in the moderate to severe range as measured by age-appropriate validated pain assessment tools. • Target lesion is uncomplicated (no fracture/spinal cord compression/cauda equina syndrome/soft tissue component) • Target lesion maximum dimension ≤3 cm (otherwise lesion may not be an OO) • Target lesion visible by noncontrast MRI • Target lesion accessible for MRgFUS procedure • MRgFUS treatment date ≥2 weeks from most recent surgical/radiologic treatment of osteoid osteoma *Exclusion criteria* • Unable to characterize pain specifically at the site of interest (target lesion) • Pregnant female • Target lesion is complicated (presence of one of fracture/soft tissue component). • Target lesion <1 cm away from the skin, neurovascular bundles, bowel, hollow viscera, or regions of cartilage/ bone growth plate • Target lesion located in the skull and spine (excluding sacrum) • Inability to position area of interest on the MRgFUS transducer • Scar along proposed MRgFUS beam path or unable to exclude scar from path • Orthopedic implant along proposed MRgFUS beam path or at site of target lesion • Serious cardiovascular, neurological, renal, or hematological chronic disease • Active infection • Contraindication to deep sedation/general anesthesia or MRI • Contraindication to gadolinium (nursing mothers, renal failure, etc.) is a relative contraindication to the procedure. MRgFUS can still be performed but perfusion information would not be obtained.	

### Diagnosis

The clinical presentation and imaging characteristics of the osteoid osteoma are assessed to help exclude common differential considerations such as a Brodie’s abscess or rare possibilities such as primary or secondary malignancy.

When a patient presents with a classic clinical history (pain, worst at night that responds to NSAIDs) and pathognomonic imaging findings, treatment can proceed in the absence of biopsy confirmation [32, 33]. When the clinical history or the imaging characteristics are in any way equivocal, a biopsy is recommended. Some operators routinely biopsy all lesions [6, 17]. When biopsy is undertaken, the access site should be chosen with the future MRgFUS trajectory in mind. Whenever possible, the resulting needle track should be perpendicular to the ultrasound treatment beam path, so that sonication through scar tissue can be avoided.

Once clinical, imaging, and/or biopsy data is reviewed, the patient, family, interventional radiologist, and orthopedic surgeon decide upon the best treatment approach. Consent to undergo treatment (and take part in any research study) is obtained from older patients and/or parents. Assent should be sought in younger patients.

### Determination of best treatment modality

Although this document focuses on guidelines and best practices for treatment of OO using MRgFUS, this modality may not be the best choice for all patients, even in light of the inclusion/exclusion criteria above. MRgFUS is a very powerful device and is changing the way some patients with OO are treated; it is still a developing research technique that has both technical limitations as well as an associated learning curve. Experience brings the ability to accurately predict when difficult lesions and complex approaches will help enable good results. During the initial development phase of an MRgFUS program, consultation with more experienced operators regarding patient eligibility, targeting, and complication avoidance when targeting complex lesions is extremely important. For the foreseeable future, surgical and/or percutaneous treatment will still be required for lesions in the spine, acoustically inaccessible locations, and MRgFUS treatment failures. The responsible physician should meet with the patient to discuss all potential options for treatment outlining the risks and benefits of each.

### Pain evaluation

The primary goal of osteoid osteoma treatment is pain relief. Easily administered validated age-appropriate measures of pain intensity must be used. The Faces Pain Scale—Revised (FPS-R) is recommended for use in children aged 4–17 years; FPS-R = 4–6 indicates moderate pain and greater than 6 severe pain [34]. The numeric rating scale (NRS) is recommended for use in adults and children >8 years; NRS = 4–6 indicates moderate pain and 7–10 severe pain [35]. The Brief Pain Inventory (BPI) is another measure of pain and pain-related disability commonly used in adults [36]. The Pediatric Initiative on Methods, Management, and Pain Assessment in Clinical Trials (PEDIMMPACT) report [37] outlines eight broad categories for reporting outcomes of pediatric pain clinical trials: pain intensity, global satisfaction with treatment, symptoms and adverse events, physical recovery/functioning (acute/chronic), emotional response/functioning (acute/chronic), role functioning, sleep, and economic factors. For future research studies, it is recommended that validated measures to capture these outcomes should also be used.

### Preoperative imaging and patient workup

As previously discussed, results of any recent existing diagnostic imaging tests should be examined to determine eligibility for MRgFUS treatment. All patients should undergo an appropriate imaging work-up to allow for diagnosis of osteoid osteoma as determined by the interventional radiologist. A standardized set of 3D MR images (see Fig. 1) will then be obtained to use for MRgFUS planning. A prior CT scan is often required to assess lesion characteristics and position.

Prior to the MRgFUS procedure, baseline blood work (complete blood count, electrolytes, creatinine) and a planning MRI are obtained. If a recent MRI is available, it can be used for treatment planning (at the discretion of the radiologist). The target lesion is assessed for any interval change (from the original imaging), and accessibility of the lesion is verified.

### Patient preparation and treatment planning

**Fig. 2:**
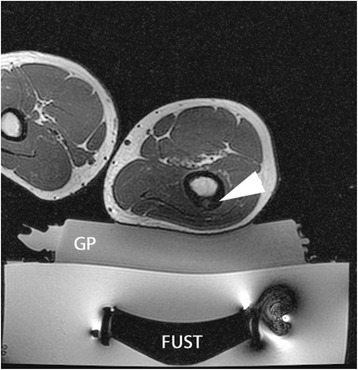
Focused ultrasound therapy setup. Axial T1-w MRI showing osteoid osteoma of right lateral femoral diaphysis (*white arrowhead*) placed onto gel pad (*GP*) overlying ultrasound transducer (*FUST*)

**Fig. 3:**
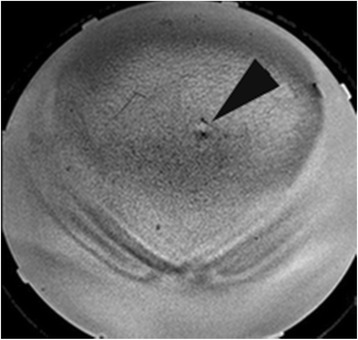
Bubble test. A coronal skin bubble scan (balanced fast gradient echo sequence) showing a 1-cm air bubble trapped between the patient and the acoustic coupling pad. If this bubble is not removed from the beam path, a skin burn could occur due to the treatment

**Fig. 4:**
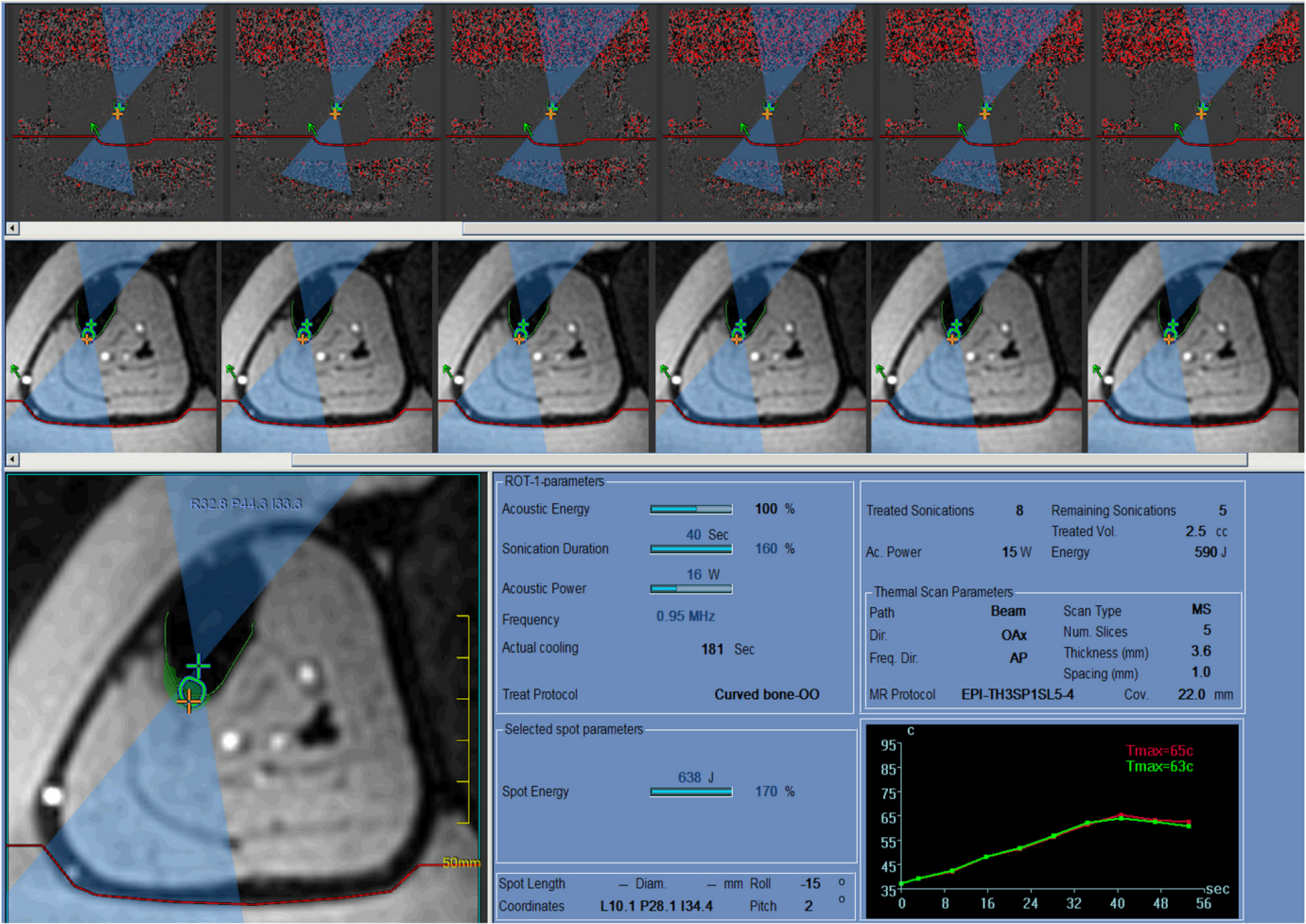
MRgFUS treatment planning. MRgFUS treatment of tibial osteoid osteoma, showing the InSightec ExAblate user interface. Subtracted phase images (*top panel*) are used to calculate temperature, with temperatures reaching 65 °C at the target (*bottom right panel*). A magnified EPI magnitude image (*bottom left panel*) shows the targeting. The position of the single focus (*green plus sign*) is adjusted such that the beam path (*blue hourglass*) intersects the bone (*green line*), creating a sonication spot (*green circle*). Thermal dose (*green*) is present at the bone surface. Note that the focal zone has been placed deep to the bone cortex in order to heat a larger bone surface area, and the beam has been oriented to avoid the neurovascular bundle

Before beginning the treatment, the following steps are performed:

- Appropriate quality assurance and safety testing of MRI and MRgFUS equipment.
- The area of interest should be shaved and a commercial depilatory cream (e.g., Nair, Veet,) may be used to further clean the skin surface. Sticky tape is used to pick up any loose hairs after shaving. The skin can be cleaned with chlorhexidine (to remove any skin oil or residue that could affect beam penetration) and allowed to dry.
- The treatment area and the corresponding skin surface for ultrasound beam entry should be landmarked.
- The patient is placed on the MRgFUS table with appropriate padding to avoid pressure-related injury and prevent movement during the procedure. The area of interest is positioned in the center of the ultrasound transducer.
- An aqueous interface (gel pad, circulating disc, water bath, etc.) will be used to couple the skin surface to the MRgFUS transducer window. All water should be degassed as per manufacturer’s specifications.
- MRI-compatible patient monitoring devices (oximetry, pulse, ECG, body temperature, etc.) are placed and enabled.
- As patients must remain still during the painful MRgFUS procedure, general anesthetic, deep sedation, local nerve blocks, and/or spinal anesthesia is used. The patient should be positioned with the area of interest on the gel pad (see Fig. 2) and then appropriately anesthetized/sedated.^1^ Any MRI imaging coils should be attached and secured at this time.
- Survey imaging and a bubble detection scan, a balanced fast field (gradient) echo sequence that is sensitive to the susceptibility artifact created at air-water interfaces, are performed (see Fig. 3). Air trapped between the transducer, gel pad, or patient could absorb or scatter the ultrasound energy and lead to unpredictable heating in the near field. If there is a bubble(s), then the patient will need to be moved off the pad, the bubble removed, the patient repositioned, and the imaging repeated.
- 3D T1- and/or T2-weighted sequences of the target bone/lesion are then acquired. These image sequences should have good bone/soft tissue/marrow contrast as well as a large enough field of view to observe the bone surface and skin entry point. If the lesion does not lie within the possible MRgFUS treatment volume, the patient is repositioned and the imaging repeated.
- This imaging sequence is then imported into the treatment planning software on the MRgFUS workstation, so that overall position of the lesion with respect to the MRgFUS device can be assessed, and the optimal acoustic window can be determined. See Fig. 4.

### Targeting, treatment execution, and monitoring

**Fig. 5:**
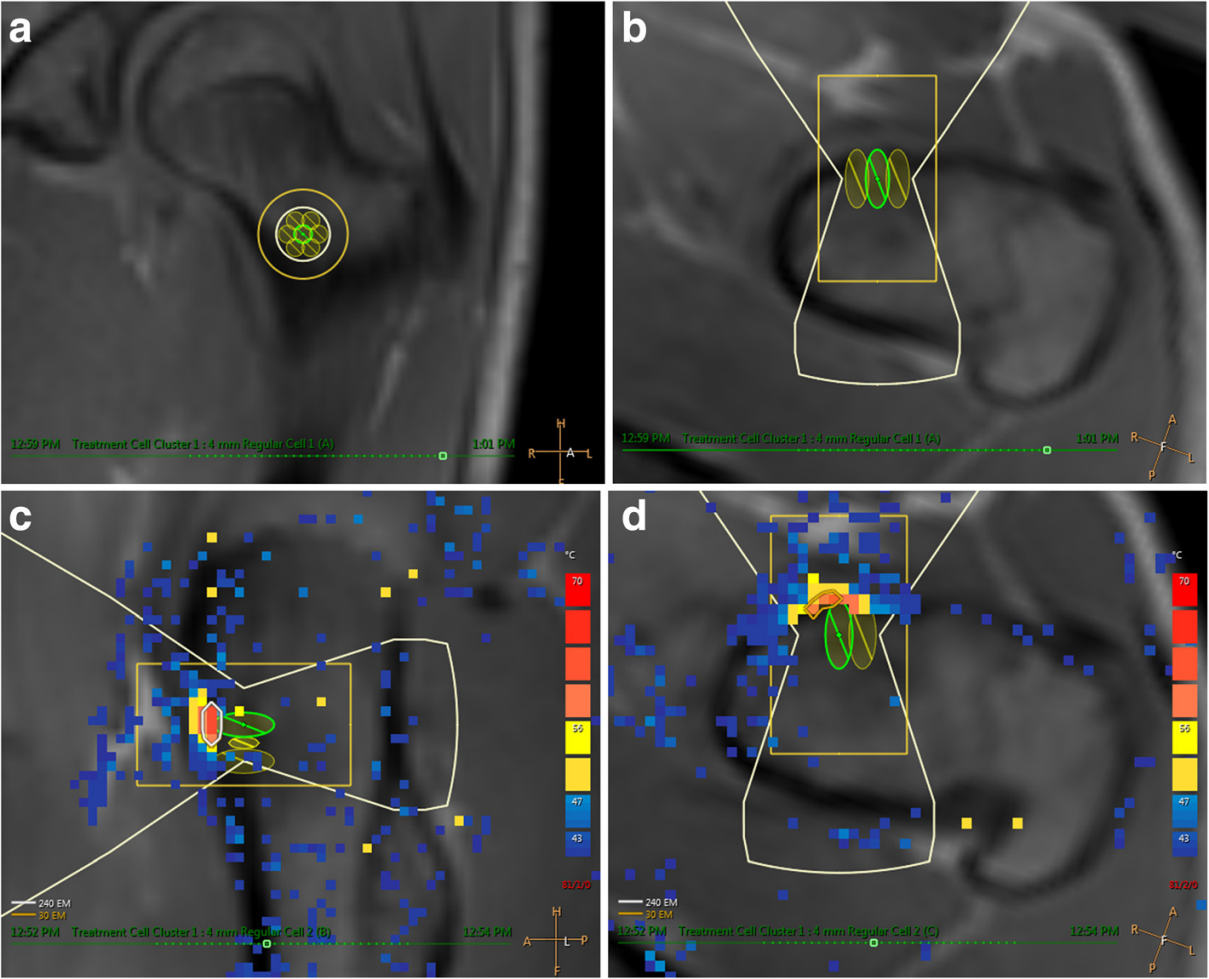
Thermography during osteoid osteoma treatment. Planned treatment cells for the osteoid osteoma from Fig. 1 shown in **a** coronal and **b** axial MRI views on a Philips Sonalleve platform. (User interface not shown). Seven 4-mm treatment cells were arranged in a circular cluster in the coronal plane to cover the entire 1-cm lesion. Due to electronic beam steering on the Sonalleve machine, the focal zone was positioned at the bone surface in the osteoid osteoma. **c** Sagittal and **d** axial images demonstrate thermal maps from a 50-W (1000 J) treatment sonication. This exposure produced a maximum temperature above 60 °C at the bone surface. A small region (approximately 1 × 4 mm) adjacent to the osteoid osteoma reached sufficient temperatures to achieve a thermal dose of 240EM@43 °C, causing necrosis.

Once all preoperative imaging has been performed and pushed to the treatment planning workstation, targeting of the OO lesion and treatment execution can begin. The following are recommended steps to execute this process:

- Positioning of the treatment cell(s) depends on the size and location of the OO and the type of equipment being used. The ultrasound focus should be placed either on the cortical surface or deep to the target lesion (e.g., between the subcortical and the far field cortex). With MRgFUS treatment of bone, it is recommended that small treatment cells are selected. Large treatment cells with electronic steering will create instances where the incident ultrasound beam is oblique to the bone surface and will be reflected or refracted to unwanted locations. An orthogonal beam trajectory will minimize the potential for off-target heating due to beam reflection/refraction.
- Depending on the size of the OO, as little as one treatment cell may be required. However, a small cluster of treatment cells placed radially around a central target maximizes energy deposition to the nidus. See Figs. 4 and 5.
- Once the treatment plan is defined, a MR thermal map sequence is prescribed. The imaging plane(s) should include the MRgFUS treatment cell as well as a near-field MRI image so that the temperature at the muscle/fat interface can be measured, which is indicative of the skin surface temperature. A far field image is not required as all the energy will be absorbed by the bone and the wave energy will not continue past the target (see Fig. 4).
- On both the InSightec ExAblate and Philips Sonalleve systems, an automated sequence completes a minimum of two pre-sonication phase image sequences (up to 3 s each), which are used to perform future subtractions for the thermal imaging sequence. To best optimize thermometry image quality, some operators suggest running the MR thermal mapping sequence for a few minutes without sonication to gauge the signal-to-noise ratio (SNR) and determine if there is any periodic shift in the image due to respiratory motion. Adjustments to the sequence (voxel size, FOV, echo time) can then be made until the background noise is acceptable.
- A test shot of 5–15 W for 10 s is performed to determine whether the acoustic energy is getting to the target bone surface or if there is undesired heating in the near field.
- Sequential treatments of 40–60 W for 20–30 s (800–1800 J) should then be performed. It is best practice to start at lower power values and progressively increase the power until the temperature measured at the target bone surface meets or exceeds 65 °C. See Figs. 4 and 5.
- The treatment session ends when all cell clusters have achieved the desired target temperature. If at any time during the treatment, near-field heating or any other unexpected behavior is observed, then the current exposure should be aborted and the treatment plan should be reevaluated. If there is concern that continued exposures will lead to an adverse event, then treatment should be aborted.
- Once the treatment concludes (or is aborted), pre- and post-T1W gadolinium sequences are acquired. Voxel-by-voxel subtraction is performed to assess posttreatment perfusion.
- The patient is removed from the MRI and recovered from anesthesia or sedation.
- Visual inspection of the skin surface is performed to assess for the presence of treatment-related skin burns.
- If any nerves were located near the treatment zone, corresponding motor and/or sensory evaluation should be performed.
- Recovered patients are either discharged or admitted for follow-up care at the discretion of the anesthesiologist and interventional radiologist.
- Patients are provided with emergency contact information and discharge instructions.
- Guidelines for ambulation following MRgFUS treatment of weight-bearing bones have not been established. Ultrasound therapy has been shown to result in bone weakening in some in vivo studies [38–40].

### Complications and complication avoidance

Although MRgFUS is a noninvasive modality for treatment of OO and other diseases, there are many considerations required to ensure patient safety and help avoid treatment complications:

- *Preparation of the skin*: One of the most effective ways to minimize a skin burn or other skin irritation is to properly shave and depilate the skin surface as mentioned above. When performed in conjunction with the use of degassed water and/or ultrasound gel for acoustic coupling of the patient to the MRgFUS system, the risk of trapped air between the patient and transducer is reduced. An MRI image of the coupling between the patient and the gel pad should be acquired to assess coupling before treatment. A balanced gradient echo image works well in this situation as air bubbles create a small susceptibility artifact. If air bubbles are discovered during this stage, the patient must be repositioned and additional degassed water should be applied to the gel pad until no bubbles are visible in the interface.
- *Location of the target*: Since bone tissue absorbs ultrasound readily and the absorption of that energy is predominately converted to heat, it is natural that bone tissue heats up more than the surrounding soft tissues. Great care must be used to ensure that a safe margin (generally considered 1 cm) of soft tissue is around the bone target before reaching the skin surface or other sensitive tissue such as the major nerves or blood vessels. For example, an osteoid osteoma located in the anterior tibia may be a poor choice for this modality as there is no mechanism for heat dissipation since there is little tissue perfusion if the skin is immediately adjacent to the bone.
- *Acoustic access to the target*: The MRgFUS transducer can analogously be compared to an optical lens, as it has a focal length and beam width. As the energy converges to the focus, the beam width narrows but may be quite wide at the entrance point into the body. Any object in the beam path that could reflect ultrasound energy, such as a gas-filled viscus or bone, needs to be outside this beam path or refocusing and off-target heating may occur. This can best be avoided by using large field of view, three plane planning images so that the entire beam path can be inspected before treatment.

### Patient follow-up and postoperative imaging

The patient should be monitored for both short- and long-term effects of treatment. The initial clinical follow-up appointment should be scheduled within the first 2 weeks and further follow-up as required for up to 6 months. As MRgFUS is a new treatment modality, follow-up MRI imaging can be considered after 3–6 months.

### Treatment failure and retreatment

There may be instances where the MRgFUS treatment of the OO does not work as effectively as expected or fails to have a therapeutic benefit. In those instances, it is important for the team to reassess what factors contributed to the less than ideal results. The cause of many treatment failures can usually be reduced to the following issues: inappropriate selection of patient, inadequate acoustic coupling, poor targeting of the lesion, and inadequate selection of treatment power and duration. These issues are summarized below:

- *Inappropriate selection of patient*: This can be a complicated issue as the thought of offering this noninvasive treatment to all patients suffering from OO can be alluring. However, restraint must be exercised to know when a patient can be better treated by another modality. Adherence to the inclusion and exclusion criteria listed above is the best way to ensure that patients are selected appropriately.
- *Inadequate acoustic coupling*: This is a two-part technical issue of MRgFUS treatment that can be avoided with care and expertise, as mentioned above.
- *Poor targeting of the lesion:* Osteoid osteomas generally occur in three distinct locations in the bone—subperiosteal, cortical, or intramedullary. Of these, subperiosteal are generally considered the easiest to treat, while intramedullary are the most challenging due to the thickness of the overlying bone. With subperiosteal lesions, a single small treatment cell can be placed directly on the target. For intramedullary lesions, the acoustic wave does not actually reach the target, as it is absorbed in the surface of the bone. For these treatments, it is the conduction of heat through the bone that leads to heating of the target. As such, the optimal position of the focus may be beyond the lesion so that a larger surface of the bone can be heated, creating a hemispherical heating aperture that converges on the lesion.
- *Inadequate selection of treatment power and duration*: The distinction between subperiosteal, cortical, and intramedullary lesions should also be considered in the selection of treatment power and duration. With subperiosteal lesions, a single exposure directly on the target with a relatively low acoustic power of 40–60 W for 20–30 s (800–1800 J) can successfully destroy the lesion. Intramedullary lesions will often require several exposures upwards of 80 W to maintain a high temperature at the bone surface, allowing time for the heat to conduct inwards into the lesion. The individual exposure times should still be kept < 40 s to allow the soft tissue to cool but since bone retains its heat longer, repeat exposures enable sustained temperature in the bone.

## Future directions

This document has outlined a series of guidelines and best practices for the thermal ablation and treatment of osteoid osteoma using MRgFUS in order to help new centers interested in this treatment approach. In addition, this could also be viewed as a first step to help organize and consolidate the clinical and research efforts of these centers to collaboratively work towards future advances in clinical MRgFUS therapies. Examples of potential future developments include:

- *Modeling*: A common feature of energy-based therapy modalities, such as radiation therapy, is the ability to model the treatment procedure and the response of tissue to treatment before the patient is actually treated. This is an emerging trend in MRgFUS treatment and active research is directed at modeling the thermal effects in and around the bone during MRgFUS treatment and estimating the response of tissue to the thermal dose. As clinical data is acquired and as these models become more accurate, a repository of clinical data could be retrospectively analyzed to validate these models using real treatment temperature data.
- *Treatment registry*: As more centers adopt this treatment approach, it will be crucial to develop patient registries to more comprehensively evaluate patient reported outcomes and facilitate clinical trials. The generation of larger data sets will help to train/test modeling techniques and create a centralized repository for all data when the community decides that dissemination of the results via peer-reviewed publication as appropriate. This data can also be used to facilitate comparisons of MRgFUS with standard treatment. For example, an MRgFUS OO treatment registry is being concurrently developed with a percutaneous OO treatment registry organized by the Society of Pediatric Interventional Radiology (SPIR).
- *Clinical trials comparing MRgFUS to conventional thermal treatments*: For MRgFUS treatment of OO to become the new standard of care, it will need to be compared to the current standard, which is radiofrequency or laser ablation. Data from the registry will facilitate design of such a trial in terms of patient numbers and outcomes and should enhance funding success rates by demonstration of a track record of collaboration.
- *Treatment of OO of the spine*: Another common location for OO is the spine, which represents one of the most difficult treatment locations using any heat-based modality, especially HIFU. Currently, the standard-of-care is percutaneous RF or laser fiber ablation since the volume of ablation and heat spread can be more confined. However, this involves guiding a bone biopsy needle into the spine. With increased experience in treating bone lesions and development of reliable thermal heading prediction models, certain areas of the spine may become viable targets.
- *Progression of equipment design*: The MR and MRgFUS equipment currently available was designed primarily for the treatment of uterine fibroids. As bone lesions can occur anywhere in the body, the MRgFUS community should work with industry partners to create equipment that is more adaptable to a wide range of patient positioning requirements.

## Conclusions

MRgFUS treatment of OO shows great promise and has numerous advantages over current therapies. This paper has provided recommendations for establishing a clinical MRgFUS treatment program and an overview of the current treatment strategy. Given the positive results of initial pilot studies, investigators should strive to organize larger multi-center studies and eventually a phase III trial comparing outcomes between MRgFUS and radiofrequency/laser ablation. Establishing MRgFUS as an effective treatment for osteoid osteoma will also help support investigation into the treatment of other bone lesions such as osteoblastoma, aneurysmal bone cyst, and eosinophilic granuloma.

## Abbreviations

BPI: Brief Pain Inventory
CT: computed tomography
FPS-R: Faces Pain Scale—Revised
MR: magnetic resonance
MRI: magnetic resonance imaging
MRgFUS: magnetic resonance-guided focused ultrasound
NRS: numeric rating scale
OO: osteoid osteoma
PEDIMMPACT: Pediatric Initiative on Methods, Management, and Pain Assessment in Clinical Trials
PGE2: prostaglandin E2
PGI2: prostacyclin or prostaglandin I2
RF: radiofrequency

## Footnotes

1 Younger or less cooperative patients may require sedation/general anesthesia prior to positioning.
